# Religiosity, Religious Fundamentalism, and Ambivalent Sexism Toward Girls and Women Among Adolescents and Young Adults Living in Germany

**DOI:** 10.3389/fpsyg.2018.02399

**Published:** 2018-12-03

**Authors:** Bettina Hannover, John Gubernath, Martin Schultze, Lysann Zander

**Affiliations:** ^1^Department of Education and Psychology, Freie Universität Berlin, Berlin, Germany; ^2^Department of Educational Science, Leibniz University Hannover, Hanover, Germany

**Keywords:** ambivalent sexism toward girls, ambivalent sexism toward women, religiosity, religious fundamentalism, right-wing authoritarianism

## Abstract

The New Year’s Eve 2015 mass sexual assaults in Germany led to a broader debate about whether the perpetrators, most of them self-identifying as Muslims, were encouraged to such acts by particularly sexist attitudes toward girls and women. Here, we argue that it is not the specific religious affiliation of individuals *per se* that predicts sexism. Rather it should be the extent to which they are involved in their religion, i.e., their religiosity and their endorsement of religious fundamentalism. In line with the theory of ambivalent sexism, we distinguish hostile and benevolent sexism, while controlling for right-wing authoritarianism and social dominance orientation. In two Pilot Studies, we explored differences in ambivalent sexism (a) between male and female individuals of Muslim faith, Christian faith, Muslim faith, Christian faith, and no religious affiliation residing in Germany, while at the same time (b) differentiating between sexism directed toward girls and sexism directed toward women. In our Main Study, we tested the interrelations between religiosity, religious fundamentalism, and ambivalent sexism in our religious subsamples of male Christians, female Christians, male Muslims, and female Muslims using a multigroup multivariate moderated mediation analysis. In all three studies, Muslims were more religious, endorsed religious fundamentalism more strongly, and held stronger benevolent sexist beliefs toward girls and women as well as stronger hostile sexist beliefs toward women than Christians and non-religious participants. In our Main Study, with female Christians as the reference group, male Muslims’ stronger benevolent and hostile sexist beliefs toward girls were mediated by religiosity and fundamentalism. Female Muslims’ stronger endorsement of benevolent sexism toward girls could be explained by their higher level of fundamentalism. While our findings show that differences in ambivalent sexism between religious groups were partly due to different levels of religiosity and fundamentalism, they also suggest that there are factors other than those investigated in our studies responsible for male Muslims’ particularly strong sexism. We discuss specific contents of Islamic religious teachings and honor beliefs as possible causes to be investigated further in future research.

## Introduction

On the last day of the year 2015 in Cologne and several other German cities (Bielefeld, Hamburg, Dortmund, Düsseldorf, Stuttgart), more than 1,000 girls and women who attended the public New Year’s Eve celebrations were sexually assaulted, mostly by groups of men suddenly surrounding and then attacking them on the street. Even adolescent girls, accompanied by their mothers or peers, were harassed. According to official estimates, at least 2,000 men were involved. Most suspects were asylum seekers and illegal immigrants from Muslim-majority countries in North Africa who had only recently arrived in Germany (Noack, 2016; see Schwarzer, 2016, for a more detailed account). A significant side effect of the New Year’s Eve mass sexual assaults was a noticeable increase of anti-Muslim sentiments in Germany (Bayrakli and Hafez, 2018).

Previous research has found sexist behavior (Begany and Milburn, 2002; Diehl et al., 2016) as well as the acceptance and occurrence of male violence against women to be predicted by sexist attitudes (Abrams et al., 2003; Chapleau et al., 2007; Koepke et al., 2014). When women deviate from traditional gender norms, they are particularly likely to become the target of hostile sexism (see Sibley and Wilson, 2004; Gaunt, 2013; Glick et al., 2015). One of the focal points of the revitalized debates on women’s rights has been the question whether the perpetrators, most of whom were practicing adherents of the religion of Islam, were encouraged in their actions by particularly sexist attitudes toward girls and women (Schwarzer, 2016).

In this article, comparing sample groups from the two monotheistic religions Christianity and Islam, we argue that it is not the specific religious affiliation but rather the extent of religious involvement, i.e., the strength of a person’s *religiosity*, and the adherence to tenets of *religious fundamentalism* that predict sexism toward girls and women. *Religiosity* here refers to the importance individuals assign to their religious beliefs (e.g., Huber and Huber, 2012), while *fundamentalism* describes the view that a set of religious teachings is infallible and the sole repository of fundamental truths. Fundamentalist believers must rigorously obey the rules of their religion in the manner that tradition has established, and those who do so are promised a special relationship with the respective deity (Kirckpatrick et al., 1991; Altemeyer and Hunsberger, 1992; Schnell, 2010). Hence, fundamentalism is not limited to any one religion but describes certain traits that can potentially be found in any religion.

As we show in more detail below, there is evidence that Muslims living in Germany describe themselves as more religious and endorse fundamentalism to a higher extent than German citizens who do not self-identify as Muslims. While most religions teach their believers that they should love and trust their fellow human beings, evidence suggests that sexism and other forms of prejudice can paradoxically be exacerbated through religion, especially in conjunction with high levels of religiosity and fundamentalism (see Hunsberger and Jackson, 2005, for a review).

We investigated this apparent paradox by inquiring into the correlation between sexism toward girls and women and the extent to which people describe themselves as more religious and/or endorse fundamentalist beliefs. We further explored whether differences in these variables could in fact be mediators of variations in the level of sexist attitudes between people of different religious affiliations by (a) investigating non-religious individuals and members of the two largest religious groups within Germany, Muslims and Christians, and (b) measuring openly negative hostile sexist attitudes and seemingly positive benevolent sexist attitudes, the two subcomponents of ambivalent sexism, as well as religiosity and fundamentalism. We then explored possible differences in ambivalent sexism (a) depending on girls versus women being the targets and (b) depending on participants’ religious affiliation and gender. In predicting sexist attitudes from religiosity and fundamentalism separately for people of different religious affiliations, we further controlled for the impact of two potential confounds present in the research literature: right-wing authoritarianism and social dominance orientation.

### Religiosity and Fundamentalism in Germany

As in many other Western countries, the percentage of people in Germany who are denominationally bound to one of the two main Christian churches is constantly declining. While in 1970 only 6.4% of the West German population had no religious affiliation, in 2011 it was 30.9% (Sachverständigenrat Deutscher Stiftungen für Integration und Migration, 2016). During the same time, due to worldwide migration, the number of people affiliated with the Muslim religion has constantly risen. The number of Muslims living in Germany rose from 500,000 in 1972 to 3 million in 2000. In 2015 it stood at 4.5 million, which corresponds to about 5% of the German population (ddp-Nachrichtenticker, 2009; REMID, 2017).

Muslim immigrants living in Germany report being more religious than German citizens who do not self-identify as Muslims (Brettfeld and Wetzels, 2007; de Hoon and van Tubergen, 2014 [investigating adolescents], Gille, 2016 [investigating adolescents]; Huber and Huber, 2012). Several studies also found that Muslims living in Germany follow the rules and traditions of their religion more strictly and more frequently engage in the requisite rituals and practices than the non-Muslim population (Albert et al., 2015 [investigating adolescents]; de Hoon and van Tubergen, 2014 [investigating adolescents]; Nyiri, 2007; Diehl et al., 2009; Fleischmann and Phalet, 2011; Diehl and Koenig, 2013).

There is also evidence that Muslims living in Germany hold more fundamentalist religious beliefs than non-Muslim German citizens. Several studies found them to more strongly endorse views that (a) only the religion of Islam (vs. Christianity for Christians) contains fundamental truth, (b) religious rules can never be changed and should be considered more important than secular law, and (c) those who do not obey them will be punished (Heitmeyer et al., 1997; Brettfeld and Wetzels, 2007; Frindte et al., 2011; Koopmans, 2015). For our own research, we therefore predicted that Muslim participants would describe themselves as more religious and endorse fundamentalist positions more strongly than Christian participants, who, in turn, would describe themselves as more religious and endorse more fundamentalist positions than participants without religious affiliation.

### Ambivalent Sexism Toward Women and Girls

The term sexism describes attitudes linked to the social category of gender which are used to preserve differences or inequality between men and women (cf. Spence, 1999; Leaper and Brown, 2017). According to ambivalent sexism theory (Glick and Fiske, 1996, 2001), sexism has both a negative and an ostensibly positive component. Hostile sexism reflects overtly negative attitudes toward girls or women marked by beliefs that they are inferior, incompetent, or trying to control men by using sex. Benevolent sexism consists of beliefs about the genders that may appear positive but are actually counterproductive to gender equity: it reflects an affectionate but patronizing attitude toward girls and women (Glick and Fiske, 1996, 2001). An instance of this can be found in the idealization of women as in need of or deserving male protection.

The concept of ambivalent sexism is useful for explaining girls’ and women’s complicity in their own subordination. Girls and women may feel privileged by being cared for and protected by men, or feel flattered by being put on a pedestal as “wonderful, pure creatures whose love is required to make a man whole” (Glick et al., 2000, p. 764). Such seeming advantages can be viewed as compensation for the disadvantages associated with hostile sexism, deceiving girls and women into perceiving the status quo gender hierarchy as fair and just and even endorsing hostile sexist beliefs (cf. Jost and Kay, 2005).

Widening the definition of sexism to include not only hostile attitudes but also ostensibly benevolent ones resolves the apparent paradox in the notion that religiosity can foster sexism. By assigning markedly different roles to men and women and justifying them as “divinely mandated,” many religions propagate ostensibly benign sexist attitudes (Glick et al., 2016, p. 547). Benevolent sexist beliefs can serve to maintain and reproduce gender inequality without making the explicit expression of negative attitudes toward girls and women a part of the religious teachings. Hence, the concept of ambivalent sexism is particularly well suited to explain the link between religiosity and sexism. In a multi-nation study, Glick et al. (2000) found that while women consistently rejected hostile sexism, the average scores of men and women on both ambivalent sexism subscales correlated quite strongly within the samples from different cultures. It seems like women are made to feel that their group is inferior to the extent to which men in their community endorse sexist beliefs. This could entail that women contribute to the maintenance of their own group’s disadvantaged status by accepting ambivalent sexism (cf. Jost and Kay, 2005). In our studies, we therefore considered it important to investigate ambivalent sexism not only in boys and men but also in girls and women.

Several studies have also applied the concept of ambivalent sexism to adolescent girls (de Lemus et al., 2008, 2010; Garaigordobil and Aliri, 2012; Ferragut et al., 2013; Montañés et al., 2013; Rau, 2013). It is in adolescence, namely when heterosexual boys typically start to anticipate or engage in intimate relations with girls, that the hostile sexist attitudes toward girls which largely prevail among boys during childhood are gradually supplemented by benevolent sexist attitudes (Glick and Hilt, 2000). Evidence for this process is provided by de Lemus et al. (2010), who found that benevolent, but not hostile, sexism toward girls increased the more experienced adolescent boys were with heterosexual romantic relationships. Aside from this study, we are not aware of research that has investigated the impact that attitudes, religious beliefs, or societal factors may have on ambivalent sexism toward girls. Since both women and girls were victimized in the events that sparked this research, we investigated each group as a potential target of ambivalent sexism to explore possible relations of ambivalent sexism toward girls with religiosity and fundamentalism.

Previous studies consistently found men to score higher than women on hostile sexism toward women. This was true irrespective of the country under investigation, as evidenced by the multi-nation study of Glick et al. (2000). Also, the gender difference in hostile sexism has been observed irrespective of whether participants identified as Jews (Gaunt, 2012), as Muslims (Taşdemir and Sakallı-Uğurlu, 2010; Glick et al., 2016), or as Christians (Glick et al., 2002; Mikołajczak and Pietrzak, 2014). In the same way, boys have consistently been found to score higher than girls on hostile sexism toward girls (de Lemus et al., 2010; Garaigordobil and Aliri, 2012; Ferragut et al., 2013; Rau, 2013). Some studies also found male participants to more strongly endorse *benevolent* sexist beliefs toward women or girls than female participants (Glick et al., 2002; Ferragut et al., 2013; Mikołajczak and Pietrzak, 2014), while others did not find a gender difference (de Lemus et al., 2010; Taşdemir and Sakallı-Uğurlu, 2010; Garaigordobil and Aliri, 2012; Gaunt, 2012; Rau, 2013; Glick et al., 2016). For our own studies, we therefore expected male participants to endorse hostile sexist beliefs toward girls and women more strongly but did not specify a directional hypothesis about gender differences in benevolent sexism.

### Religiosity and Ambivalent Sexism in Different Religious Groups

Research shows that religiosity is associated with gender inequality (e.g., Klingorová, 2015), sexism, and negative attitudes toward gender equality (e.g., Diehl et al., 2009; Seguino, 2011; Adamczyk, 2013). Using data from the World Values Survey, for example, Adamczyk (2013) found that the more religious participants described themselves to be, the more they endorsed gender inequality. Surveying 4,000 Turks living in Germany (90% of them identifying as Muslims) and 10,000 Germans with no migration background (70% self-identifying as Christians), Diehl et al. (2009) found that high religiosity was negatively related to the approval of gender equality in both groups, even after controlling for education and employment status.

Empirical findings regarding the relation between religiosity and ambivalent sexism are less clear-cut. While higher levels of religiosity have been consistently related to a stronger prevalence of benevolent sexism, evidence has been mixed for the association between religiosity and hostile sexism. In a convenience sample of Jewish participants from Israel, Gaunt (2012) found positive relations between religiosity and benevolent, but not hostile, sexism toward women. In a sample of more than 1,000 men and women from Spain, Glick et al. (2002) found that Catholic religiosity predicted stronger benevolent, but not hostile, sexism toward women. Similarly, Mikołajczak and Pietrzak (2014), investigating a convenience sample from Poland, found Catholic religiosity to covary with benevolent, but not hostile, sexism toward women. In a sample of Evangelical Christian undergraduate students from the United States, Maltby et al. (2010) found Christian orthodoxy to correlate with one of the three subfactors of benevolent sexism toward women, protective paternalism, in men but not in women. In contrast, no relation was found between Christian orthodox beliefs and hostile sexism.

The two studies we are aware of which did find interrelations between religiosity and hostile sexism investigated undergraduate students from Turkey. Taşdemir and Sakallı-Uğurlu (2010) and Glick et al. (2016) found positive correlations between Muslim religiosity and both subtypes of ambivalent sexism toward women. We are not aware of any study investigating religiosity and ambivalent sexism toward girls.

Taken together, this pattern of findings is consistent with the view that benevolent sexism toward women is tolerated or even encouraged by various religions, while hostile sexism seems to be absent from all the religions investigated aside from Islam (cf. Hunsberger and Jackson, 2005; Whitley, 2009). However, none of the cited studies have accounted for the potential influence of fundamentalism and other ideologies favoring outgroup-derogation, such as right-wing authoritarianism or social dominance orientation. For our own research, we therefore hypothesized that interrelations between religiosity and ambivalent sexism would be attenuated if these confounders were accounted for, and thus included them in our investigations. Since, to our knowledge, no research has yet investigated religiosity and fundamentalism as predictors of ambivalent sexism toward girls, we refrained from formulating a directional hypothesis specifying differences based on whether girls or women are the targets of sexism.

When comparing previous studies investigating people of varying religious affiliations in different countries, the mean values obtained for benevolent and hostile sexism toward women were higher in samples of Muslims (Taşdemir and Sakallı-Uğurlu, 2010; Glick et al., 2016) than in samples of Christians (Glick et al., 2002; Maltby et al., 2010; Mikołajczak and Pietrzak, 2014). We did not find any studies comparing the levels of ambivalent sexism toward girls in different religious groups. Also, no previous study has investigated a religious group that represents a minority in the respective country. According to traditional acculturation theories, religious minority groups can be expected over time to become increasingly similar in their beliefs to the religious majority (cf. Alba and Nee, 1997). Yet, minority status can trigger reactivity as well, i.e., a contrasting of personal beliefs from the ones shared by the majority (e.g., Diehl and Koenig, 2013). Hence, it is plausible for Muslims residing in Germany to be either less sexist or more sexist than fellow believers living in countries with a Muslim majority. In order to analyze whether potential differences are mediated by differences in religiosity and fundamentalism in our Main Study, we ran two Pilot Studies exploring possible differences in ambivalent sexism between religious groups.

### Religiosity and Fundamentalism as Predictors of Sexism

Many studies have identified a link between fundamentalism and negative attitudes, or open hostility, toward outgroups. While most studies examining the fundamentalism-prejudice link have investigated negative attitudes toward minority groups, such as homosexuals (Whitley, 2009, for a review), transgender individuals (e.g., Nagoshi et al., 2008), or racial minorities (Hall et al., 2010, for a review), only a few have also looked at gender-related prejudice (attitudes toward women: McFarland, 1989; Hunsberger et al., 1999; endorsement of rape myth: Sheldon and Parent, 2002; ambivalent sexism: Hill et al., 2010).

A closer look at the interrelatedness of fundamentalism, religiosity, and negative attitudes toward outgroups suggests that the religiosity-sexism link can be at least partly explained by fundamentalism. For instance, in a sample from the United States consisting of undergraduates Johnson et al. (2011) found that fundamentalism strongly covaried with religiosity and, together with right-wing authoritarianism, mediated the relation between religiosity and negative prejudice against homosexuals or African Americans. Similarly, in a European multi-national study Koopmans (2015) found that fundamentalism was strongly related to out-group hostility, while religiosity, controlling for the impact of fundamentalism, was not. Further, Kirckpatrick et al. (1991) and Altemeyer and Hunsberger (1992), investigating college students from the United States and Canada, found that religiosity was unrelated to discriminatory attitudes toward various minority groups once fundamentalism had been controlled for.

Fundamentalism has been found (Banyasz et al., 2016; Harnish et al., 2017) to be strongly correlated with both social dominance orientation (SDO; Pratto et al., 1994) and right-wing authoritarianism (RWA; Altemeyer, 1996). One plausible explanation is that all three ideologies are associated with cognitively rigid thinking (cf. Hill et al., 2010; Brandt and Reyna, 2014). SDO is based on the belief that some groups are superior to others, a belief that coincides with endorsing the suppression of outgroups and a preference for hierarchy within any social system. RWA is a social ideology favoring traditional values and obedience to authority figures, composed of three attitudinal clusters: authoritarian submission, authoritarian aggression, and conventionalism. The Religious Fundamentalism-Scale (we used the German version by Schnell, 2010) developed by Altemeyer and Hunsberger (1992, 2004), for example, has determined strong associations between fundamentalism and RWA (for a review see Altemeyer and Hunsberger, 2004: correlations between 0.62 and 0.82). Also, Sibley et al. (2007) found that RWA and SDO correlated with both benevolent and hostile sexism toward women. We therefore included measures of RWA and SDO to account for these potential confounding variables. To avoid suppression effects and statistical artifacts (Mavor et al., 2009), we treated them as controls in the regression analyses of our Main Study.

### The Present Research

In light of relevant findings by previous research, we expected (a) that male participants would show more hostile sexism (but not necessarily more benevolent sexism) toward girls and women than female participants would, and (b) that Muslims would score highest on religiosity and fundamentalism, followed by Christians and, lastly, non-religious individuals. To test our research instruments and determine whether we would need to take differences in ambivalent sexism between religious groups into account, we ran two Pilot Studies.

The core assumption of our research was tested in our Main Study with a multigroup multivariate moderated mediation analysis. We expected that religiosity and fundamentalism would be associated with ambivalent sexism irrespective of religious affiliation, but that potential group differences in ambivalent sexism would, at least partly, be mediated by differences in levels of religiosity, fundamentalism, RWA, and SDO.

## Materials and Methods

All surveys were conducted with the informed consent of each participant. More specifically, participants were informed that (1) this research was being conducted by researchers from Freie Universität Berlin, (2) the purpose of the research was to investigate adolescents’ and adults’ values and attitudes toward life, (3) the expected duration would be about 5 min, (3) they had the right to withdraw from the research at any point after participation had begun, (4) there was no financial inducement for participation, and (5) no information relating to the person’s identity, such as their name, email or home address would be collected. They were further informed whom to contact for questions about the research (Pilot Study 1, Main Study) or provided opportunity to ask questions and receive answers from the interviewers (Pilot Study 2, Main Study).

### Research Instruments

Religiosity (Pilot Studies 1, 2, Main Study) was measured via the German version of the Centrality of Religiosity Scale (CRS, Huber and Huber, 2012), which is suitable for at least the Abrahamic religions (Judaism, Christianity, Islam). With 15 items, the scale measures the centrality or importance the participant attaches to religious beliefs (e.g., “How often do you take part in religious services?”, “How often do you experience situations in which you have the feeling that God or something divine intervenes in your life?”). All answering scales provided five options that referred either to frequency (1 = never, 2 = seldom, 3 = sometimes, 4 = often, very often) or intensity (1 = not at all, 2 = rather not, 3 = somewhat, 4 = rather yes, 5 = very much so), depending on the content of the item.

Fundamentalism (Pilot Studies 1, 2, Main Study) was measured with the Innsbrucker Religiöser-Fundamentalismus-Skala (IRFS, Schnell, 2010), a shortened and adapted German version of the Religious Fundamentalism Scale by Altemeyer and Hunsberger (1992), revised 2004). With eight items and five-point answering scales (1 = strongly disagree, 5 = strongly agree), the one-dimensional scale grasps the extent to which individuals believe that the traditions of their religion are inerrant (e.g., “The traditions and scripts of my religion are without error.”), binding and beyond question (e.g., “Someone who compromises the traditions of religion cannot be a true follower of God.”), and lead to a special relation with God for those who adhere to the rules they establish (e.g., “Only those who fully comply with the rules of my religion will experience happiness and salvation”).

Ambivalent sexism toward girls (Pilot Study 1, Main Study) was measured with the Ambivalent Sexism toward Girls in Adolescents Inventory (Rau, 2013), a German version of the Ambivalent Sexism Inventory (ASI, Glick and Fiske, 1996) adapted for adolescents. The inventory uses five-point response scales (1 = strongly disagree, 5 = strongly agree), with 12 items relating to hostile sexism (e.g., “In a group, a boy is the better leader,” “Girls are difficult to predict: they constantly change their minds.”) and 13 items relating to benevolent sexism (e.g., “If a girl feels cold, the boy should give her his sweater even if he feels cold himself,” “Girls care more about others than boys do”).

Ambivalent sexism toward women (Pilot Study 2) was measured with six items from the German version of the ASI by Eckes and Six-Materna (1999) pertaining to hostile sexism (e.g., “Most women fail to appreciate fully all that men do for them”), and six items pertaining to benevolent sexism (e.g., “In a disaster, women ought to be rescued before men”, response scales: 1 = strongly disagree – 6 = strongly agree).

Right-wing authoritarianism (Pilot Study 2, Main Study) was measured with six items taken from the German short version of the scale by Altemeyer (1996) developed by Beierlein et al. (2014; sample item: “What we really need are strong, determined leaders, to live securely in our society,” answering scales: 1 = strongly disagree to 5 = strongly agree).

Social dominance orientation (Pilot Study 2, Main Study) was measured with eight items (e.g., “We should do what we can to equalize conditions for different groups,” answering scales: 1 = strongly disagree to 5 = strongly agree) taken from the scale by Carvacho et al. (in preparation), a short version of the scale by Ho et al. (2015) translated into German.

### Statistical Analyses

To investigate possible differences between genders and religious groups concerning levels of religiosity, fundamentalism, and ambivalent sexism, we conducted, whenever admissible and unless otherwise stated, two-factorial (religious group, gender) ANOVAs (Pilot Studies 1, 2, Main Study). Since heteroscedasticity was plausible, for example, for religiosity between non-religious and religious participants, the HC3 approach described by MacKinnon and White (1985) implemented in the *car*-Package (Fox and Weisberg, 2011) for *R* (R Core Team, 2017) was applied in accordance with the recommendations of Long and Ervin (2000). Accordingly, *post hoc* group comparisons were performed using *t*-Tests with Welch-corrected degrees of freedom. When Shapiro–Wilk tests indicated deviations from the assumption of normality for any of the investigated groups after Bonferroni–Holm adjustment, median-based tests as described and recommended by Wilcox (2012) and implemented in the *WRS2*-package (Mair et al., 2017) for *R* were used as a robust alternative to classical ANOVAs. Multiple comparisons and inference regarding correlations were corrected using the Bonferroni–Holm adjustment (Holm, 1979). In our Main Study, we investigated our core hypothesis regarding the predictability of ambivalent sexism from religiosity and fundamentalism by estimating a multigroup moderated mediation analysis.

### Pilot Study 1

Our first goal was to examine ambivalent sexism toward girls and identify differences according to gender and religious affiliation. To do so, we conducted an online survey using QuestBack GmbH’s online surveying platform Unipark. Since we targeted adolescents and young adults, the survey was primarily shared on the social network platform Facebook and in online forums for religious adolescents ^1,2,3^). Additional adolescents were recruited via e-mail distribution lists of religious youth clubs.

#### Research Participants

We reached 132 adolescents and young adults (50 male, 60 female, 22 missing) between 12 and 32 years of age (*M*_age_ = 19.36, *SD* = 3.82). Fifty-six participants self-identified as Christians, 15 as Muslims, 28 as not having any religious affiliation, and 11 as having a religious affiliation other than Christian or Muslim (22 missing). Only participants of Christian or Muslim faith, as well as non-religious participants, were included in the subsequent analyses, reducing the sample size to 99 (43 male, 56 female).

#### Research Instruments

The following reliabilities were obtained for the scales administered in Pilot Study 1: Centrality of Religiosity Scale (α = 0.97), Innsbrucker Religiöser-Fundamentalismus-Scale (α = 0.93), and the Ambivalent Sexism toward Girls in Adolescents Inventory (hostile sexism: α = 0.90; benevolent sexism: α = 0.86).

#### Results

Table 1 depicts the results of ANOVAs or, where deviations from the assumption of normality had been observed, ANOVAs for medians, conducted on religiosity, fundamentalism, and sexism toward girls. Means and standard deviations are reported in the Supplementary Table S1 in the Appendix. In the following, we only report statistically significant findings.

**Table 1:** Main effects and interaction effects from ANOVAs (*F*-values)/ANOVAs for medians (*V*-values) on religiosity, fundamentalism, benevolent and hostile sexism toward girls in Pilot Study 1.

	Main effect Religious affiliation	Main effect Gender	Interaction effect Religion × Gender
Religiosity	*F*[2,93] = 110.11, *p* < 0.001^a,b,c^	*F*[1,93] = 0.02, *p* = 0.877	*F*[2,93] = 0.22, *p* = 0.802
Fundamentalism	*V*[2, ∞] = 33.32, *p* < 0.001^a,b,c^	*V*[1, ∞] = 0.37, *p* = 0.546	*V*[2] = 3.15, *p* = 0.207
Benevolent sexism	*F*[2,93] = 8.30, *p* < 0.001^b,c^	*F*[1,93] = 22.88, *p* < 0.001	*F*[2,93] = 0.07, *p* = 0.936
Hostile sexism	*F*[2,93] = 1.38, *p* = 0.257	*F*[1,93] = 2.99, *p* = 0.087	*F*[1,93] = 0.02, *p* = 0.983

*Superscripted letters indicate which of the three post hoc group comparisons for the main effect for religious affiliation are significant at p < 0.05 after Bonferroni–Holm correction: a: Non-religious – Christian, b: Non-religious – Muslim, c: Christian – Muslim*.

Participants who identified as Muslims were more religious (*M* = 4.54, *SD* = 0.28) than Christians (*M* = 3.44, *SD* = 1.01) and non-religious participants (*M* = 1.95, *SD* = 0.87). Muslim respondents endorsed fundamentalism to a stronger extent (*M* = 4.08, *SD* = 0.54) than Christians (*M* = 2.40, *SD* = 1.12) and non-religious participants (*M* = 1.71, *SD* = 0.84).

Regarding benevolent sexist beliefs toward girls, Muslim participants endorsed them more strongly (*M* = 3.57, *SD* = 0.66) than Christian participants (*M* = 2.89, *SD* = 0.76) and non-religious participants (*M* = 2.87, *SD* = 0.87). Male participants showed higher levels of benevolent sexism than female participants (*M*_male_ = 3.41, *SD*_male_ = 0.69; *M*_female_ = 2.67, *SD*_female_ = 0.75). No significant effects were observed for hostile sexism toward girls.

Table 2 depicts correlations between all measured variables. Participants held more benevolent sexist attitudes toward girls the more religious they described themselves to be and the more they reported accepting religious fundamentalist beliefs. Hostile sexism covaried with fundamentalism, while the association with religiosity was not statistically significant. As our subsample of Muslim participants was extremely small (*n* = 15), we refrained from calculating separate correlation coefficients according to religious affiliation.

**Table 2:** Correlations among religiosity, fundamentalism, benevolent and hostile sexism toward girls in Pilot Study 1.

	Religiosity	Fundamentalism	Benevolent sexism
Religiosity	–		
Fundamentalism	0.76***	–	
Benevolent sexism	0.27*	0.35**	–
Hostile sexism	0.19	0.32**	0.70^∗∗∗^

*N = 101. ^∗^p < 0.05, ^∗∗^p < 0.01, ^∗∗∗^ p < 0.001*.

### Pilot Study 2

Our next goal was to investigate ambivalent sexism toward women. Again, we explored differences according to gender and religious affiliation.

#### Research Participants

In four different neighborhoods of a large German city, teams of one female and one male psychology student approached passersby in public places (e.g., shopping areas, children’s playgrounds) and asked them to fill out our questionnaire. In doing so, we reached a sample of 146 adolescents and adults (71 women, 73 men, 2 indicated a different gender) between 13 and 77 years (*M* = 34.43, *SD* = 13.84). Muslims participants were significantly younger (*Mdn* = 26) than Christian (*Mdn* = 34, *H* = 13.67, *p* < 0.001) and non-religious participants (*Mdn* = 32, *H* = 12.47, *p* < 0.001). Fifty-three participants self-identified as non-religious, 34 as Christians, and 48 as Muslims (7 other religious affiliations, 4 missing). Only Christians, Muslims, and non-religious participants who indicated their gender were included in subsequent analyses (*N* = 134).

#### Research Instruments

Reliability for the ambivalent sexism toward women scale was very good (hostile sexism: α = 0.87; benevolent sexism: α = 0.87). As we had asked our research participants to fill out our questionnaire on the street, it was important that it could be completed within a few minutes. To ensure this, we shortened the scale measuring religiosity to six items (α = 0.94) and the scale measuring fundamentalism to five items (α = 0.93). Pilot Study 2 additionally included the construal variables RWA (showing an acceptable reliability: α = 0.80) and SDO (displaying a mediocre, but still acceptable reliability: α = 0.67).

#### Results

Table 3 displays the results of ANOVAs or, where deviations from the assumption of normality had been observed, ANOVAs for medians, conducted on religiosity, fundamentalism, sexism toward women, RWA, and SDO. Means and standard deviations are reported in the Supplementary Table S2 in the Appendix. Only significant effects will be described in the following.

**Table 3:** Main effects and interaction effects from ANOVAs (*F*-values)/ANOVAs for medians (*V*-values) on religiosity, fundamentalism, benevolent and hostile sexism toward women, right-wing authoritarianism, and social dominance orientation in Pilot Study 2.

	Main effect Religious Affiliation	Main effect Gender	Interaction effect Religion × Gender
Religiosity	*V*[2,∞] = 64.78, *p* < 0.001^a,b,c^	*V*[1,∞] = 0.01, *p* = 0.931	*V*[2] = 21.67, *p* < 0.001
Fundamentalism	*V*[2,∞] = 38.81, *p* < 0.001^a,b,c^	*V*[1,∞] = 0.78, *p* = 0.377	*V*[2] = 3.66, *p* = 0.160
Benevolent sexism	*F*[2,128] = 29.58, *p* < 0.001^b,c^	*F*[1,128] = 0.95, *p* = 0.332	*F*[2,128] = 1.14, *p* = 0.324
Hostile sexism	*V*[2,∞] = 18.01, *p* < 0.001^b,c^	*V*[1,∞] = 0.55, *p* = 0.459	*V*[2] = 5.59, *p* = 0.061
Right-wing authoritarianism	*F*[2,325] = 15.10, *p* < 0.001^b,c^	*F*[1,325] = 2.12, *p* = 0.146	*F*[2,325] = 2.76, *p* = 0.065
Social dominance orientation	*V*[2,∞] = 0.00, *p* = 0.999	*V*[1,∞] = 0.21, *p* = 0.649	*V*[2] = 3.39, *p* = 0.184

*Superscripted letters indicate which of the three post hoc group comparisons for the main effect for religious affiliation are significant at p < 0.05 after Bonferroni–Holm correction*: *a: Non-religious – Christian, b: Non-religious – Muslim, c: Christian – Muslim*.

Participants of Muslim faith described themselves as significantly more religious (*M* = 3.72, *SD* = 0.92) than Christians (*M* = 2.51, *SD* = 1.23) and non-religious participants (*M* = 1.57, *SD* = 0.71). Muslims endorsed fundamentalism (*M* = 3.50, *SD* = 1.25) to a stronger extent than Christians (*M* = 1.65, *SD* = 0.95), and Christians endorsed it more strongly than non-religious participants (*M* = 1.21, *SD* = 0.39).

Muslims endorsed benevolent sexist beliefs toward women more strongly (*M* = 4.36, *SD* = 1.20) than the other two groups (*M*_Christians_ = 2.85, *SD* = 1.21; *M*_non-religious_ = 2.54, *SD* = 1.12). Also, Muslim participants (*M* = 3.38, *SD* = 1.12) endorsed more hostile sexist positions toward women than the other groups (*M*_Christians_ = 2.01, *SD* = 0.96; *M*_non-religious_ = 1.91, *SD* = 0.91). Further, Muslim participants indicated higher levels of support for RWA (*M* = 3.09, *SD* = 0.90) and SDO (*M* = 2.49, *SD* = 0.75) than the other groups (Christians: *M*_RWA_ = 2.09, *SD* = 0.74; *M*_SDO_ = 1.83, *SD* = 0.66; non-religious participants: *M*_RWA_ = 1.94, *SD* = 0.70; *M*_SDO_ = 1.78, *SD* = 0.59).

Table 4 depicts correlations between all measured variables for the entire sample, separated by religious affiliation. Participants held more benevolent sexist attitudes the more religious they described themselves as being, and the more they accepted fundamentalist tenets. For hostile sexism, the pattern and magnitude of correlations were similar. Calculated within the religious groups of Christians and Muslims, as shown in Table 4, all correlations between our religion- and sexism-related variables were positive. However, they varied in strength and many of them did not reach statistical significance.

**Table 4:** Correlations among religiosity, fundamentalism, benevolent and hostile sexism toward women, right-wing authoritarianism, and social dominance orientation in Pilot Study 2.

	Religiosity	Fundamentalism	Benevolent sexism	Hostile sexism	Right-wing authoritarianism
Religiosity	–				

Fundamentalism	0.74^∗∗∗^ (0.38 / 0.70^∗∗∗^ / 0.46)	–			

Benevolent sexism	0.49^∗∗∗^ (-0.26 / 0.47 / 0.21)	0.64^∗∗∗^ (0.00 / 0.37 / 0.53^∗∗^)	–		

Hostile sexism	0.56^∗∗∗^ (0.21 / 0.44 / 0.27)	0.65^∗∗∗^ (0.46^∗^ / 0.64^∗∗^ / 0.38)	0.76^∗∗∗^ (0.65^∗∗∗^ / 0.64^∗∗∗^ / 0.63^∗∗∗^)	–	

Right-wing authoritarianism	0.50^∗∗∗^ (-0.02 / 0.30 / 0.27)	0.72^∗∗∗^ (0.34 / 0.39 / 0.72^∗∗∗^)	0.73^∗∗∗^ (0.64^∗∗∗^ / 0.39 / 0.67^∗∗∗^)	0.69^∗∗∗^ (0.67^∗∗∗^ / 0.48 / 0.48^∗^)	–

Social dominance orientation	0.38^∗∗∗^ (0.11 / 0.07/ 0.20)	0.53^∗∗∗^ (0.47^∗^ / -0.15 / 0.30)	0.34^∗∗∗^ (0.06 / -0.15 / 0.30)	0.47^∗∗∗^ (0.36 / 0.20 / 0.27)	0.47^∗∗∗^ (0.25 / 0.17 / 0.37)

*Correlations for each religious group are shown in parentheses in the order non-religious / Christian / Muslim. Due to occasional missing data, Ns range as follows: 128 – 136 (51 – 54 / 33 – 36 / 48 – 50). p-values were Bonferroni–Holm corrected within each group but not across groups. ^∗^p < 0.05, ^∗∗^p < 0.01, ^∗∗∗^p < 0.001*

### Discussion of Pilot Studies 1 and 2

Due to the small sample sizes, we refrained from conducting more complex analyses which would have allowed us to control for potential confounders. While in both Pilot Studies Muslims described themselves as more religious and fundamentalist than the two other groups, floor effects were observed in the statistical distribution of religiosity among non-religious participants and of fundamentalism among Christians and non-religious participants. We therefore oversampled religious participants, particularly of Muslim but also of Christian faith in our Main Study.

In Pilot Study 1, we found higher levels of benevolent sexism toward girls among Muslims than in the other two groups. There were no differences in hostile sexism toward girls. In Pilot Study 2, Muslims endorsed both benevolent and hostile sexist beliefs toward women more strongly than Christians and non-religious participants, while the latter two groups did not differ from one another.

With respect to gender, both Pilot Studies showed, contrary to our expectation, that male and female participants did not differ in their levels of hostile sexism toward girls or women. While male participants showed higher levels of benevolent sexism toward girls than female participants in Pilot Study 1, there was no such difference in benevolent sexism toward women in Pilot Study 2. We aimed to clarify these partly unexpected findings in the investigation of sexism toward girls in our Main Study.

In our Pilot Studies, we found small- to medium-sized correlation coefficients between the religiosity-related variables and ambivalent sexism when investigating the overall samples. These correlations may, however, be at least partly due to mean differences between religious groups for both types of variables. When considered within the religious groups in Pilot Study 2, correlation coefficients varied in strength and were statistically non-significant in many cases.

Our Main Study therefore aimed at investigating whether the differences between genders and religious groups that we uncovered in ambivalent sexism were (at least partly) due to group differences in religiosity and fundamentalism. Additionally, because interrelations between the variables varied considerably across the groups, we conducted a moderated mediation analysis to investigate the links in each of our religious groups of Muslims and Christians independently.

### Main Study

#### Research Participants

For our Main Study, we enhanced our efforts to reach Muslims and Christians not only by launching an internet-based online survey via Unipark, but also by systematically approaching potential participants in places where we expected to find younger religious people (e.g., youth clubs in particular districts of a large German city). As it turned out to be very difficult to gain religious participants, particularly so for boys and young men, we loosened the age-related criterion we had applied in Pilot Study 1 and also approached people of middle age. As in Pilot Study 2, the face-to-face interviews were conducted by teams of one male and one female student.

Altogether, 350 people between 13 and 48 years (*M* = 21.31, *SD* = 4.92) participated (127 male, 221 female, 2 missing). Of those, 166 were assessed via an online questionnaire and 184 via interview. Forty-three participants were non-religious, 106 Christians, and 191 Muslims. Ten participants were of a different religion and excluded. Muslim participants (*Mdn* = 21) were younger than non-religious participants (*Mdn* = 24, *H* = 6.50, *p* = 0.022) and Christian participants (*Mdn* = 22, *H* = 7.98, *p* = 0.014). Non-religious participants did not differ in their mean age from Christians (*H* = 1.55, *p* = 0.214). While gender was relatively balanced within the Muslim group (97 female, 94 male), the sample of non-religious participants was somewhat (30 female, 13 male), and the sample of Christians highly (91 female, 15 male) skewed toward female participants.

#### Research Instruments

Our Main Study used exactly the same scales as in Pilot Study 1, supplemented by the measures of RWA and SDO already applied in Pilot Study 2. The scales reached the following reliabilities in our Main Study: the complete version of the religiosity scale (α = 0.96), the complete version of the fundamentalism scale (α = 0.95), the Ambivalent Sexism toward Girls Inventory (benevolent sexism: α = 0.87; hostile sexism: α = 0.96), the RWA-scale (α = 0.82), and the SDO-scale (α = 0.79).

#### Statistical Analyses

We again conducted ANOVAs and ANOVAs for medians to detect group differences according to religious affiliation (Muslims, Christians, non-religious participants) and gender regarding ambivalent sexism, religiosity, fundamentalism, SDO, and RWA.

To investigate our main hypotheses regarding the predictability of benevolent and hostile sexism from the religion-related variables, only participants reporting to be either of Christian or Muslim faith were included in the following analyses, resulting in four gender-religion combination groups. In a first step, we estimated a multigroup multivariate regression using lavaan (Version 0.5-23.1097; Rosseel, 2012), with both forms of sexism simultaneously included as outcomes. Since we found correlations between the variables to differ according to participants’ religious affiliation in Pilot Study 2, we estimated the regression weights freely, meaning that they were allowed to differ across the four groups.

This procedure resulted in a model in which the influences of the five predictors (religion, fundamentalism, RWA, SDO, and age) on the two forms of sexism (hostile, benevolent) are assumed to be moderated by the grouping variable (i.e., the religious affiliation-gender combination). To determine whether the group differences in religiosity and fundamentalism identified in Pilot Studies 1 and 2 were associated with the group differences we identified for ambivalent sexism, we also tested the pathways for mediation. In line with Muthén and Asparouhov (2015), this allows for the identification of three separate effects.

(1) The total natural indirect effect (TNIE) represents the overall influence of the difference between groups in the outcome that is mediated via the intermediate variables. Hence, the TNIE depicts differences between, for example, female Christians and male Muslims in ambivalent sexism that can be explained by the differences between these two groups in religiosity, religious fundamentalism, RWA, SDO, and age.

(2) The pure natural direct effect (PNDE) represents the group differences in ambivalent sexism that go beyond the mediated components, meaning, for example, that female Christians and male Muslims differ in ambivalent sexism due to pathways not captured in the variables assessed in this study.

(3) The total effect (TE) constitutes the sum of the former two, thus representing the overall influence of group differences on the outcomes, that is the overall difference in ambivalent sexism between, for example, female Christians and male Muslims.

To test these effects, we applied the Monte Carlo resampling methods described by Tofighi and MacKinnon (2016), and implemented in the R package RMediation by^4^ Tofighi and MacKinnon (2011). We did so because the bootstrap resampling methods which are often applied in these situations have performed poorly in small samples (Koopman et al., 2015). As Christians are the majority religious group in Germany, they are suitable as a reference in the analyses. Since the share of male Christians was very small in our sample, we selected female Christians as the reference group. We tested the bivariate normality of both types of sexism using the Mardia Test as implemented in the psych package for R (Revelle, 2018) and found significant skew in all four groups included in the model. To accommodate the non-normality of the variables, we chose robust standard errors via the MLR estimator implemented in lavaan.

#### Results

Means and standard deviations for the following ANOVAs are depicted in Table 5. Table 6 displays the results of ANOVAs or, where deviations from the assumption of normality had been observed, of ANOVAs for medians, conducted on religiosity, fundamentalism, sexism toward women, RWA, and SDO in our Main Study.

**Table 5:** Means and standard deviations (in parentheses) for religiosity, fundamentalism, benevolent and hostile sexism toward girls, right-wing authoritarianism, and social dominance orientation separated by religious groups and participant gender in Main Study.

	Non-religious			Christian			Muslim		
	Female (*n* = 30)	Male (*n* = 13)	Overall (*n* = 43)	Female (*n* = 91)	Male (*n* = 15)	Overall (*n* = 106)	Female (*n* = 96)	Male (*n* = 91)	Overall (*n* = 187)
Religiosity	1.69 (0.48)	1.80 (0.61)	1.72 (0.52)	2.92 (0.87)	2.70 (0.92)	2.89 (0.88)	3.82 (0.75)	3.69 (0.69)	3.76 (0.72)
Fundamentalism	1.32 (0.65)	1.46 (0.76)	1.36 (0.67)	1.58 (0.60)	1.38 (0.55)	1.55 (0.60)	3.08 (0.92)	3.71 (0.97)	3.39 (0.99)
Benevolent sexism	2.21 (0.70)	2.31 (0.65)	2.24 (0.68)	2.36 (0.63)	2.74 (0.71)	2.41 (0.65)	2.84 (0.81)	3.13 (0.67)	2.98 (0.76)
Hostile sexism	1.45 (0.51)	1.89 (1.09)	1.58 (0.75)	1.58 (0.48)	1.99 (0.96)	1.64 (0.58)	1.64 (0.59)	3.40 (0.96)	2.52 (1.19)
Right-wing authoritarianism	2.15 (1.00)	2.12 (0.79)	2.14 (0.93)	2.08 (0.65)	2.17 (0.97)	2.10 (0.70)	2.47 (0.67)	3.02 (0.73)	2.74 (0.75)
Social dominance orientation	3.42 (0.37)	3.41 (0.39)	3.42 (0.37)	3.34 (0.45)	3.44 (0.37)	3.35 (0.44)	3.38 (0.37)	3.36 (0.42)	3.37 (0.39)

*All items were measured on 1–5 response scales, with higher values indicating stronger endorsement.*

**Table 6:** Main effects and interaction effects from ANOVAs (*F*-values)/ANOVAs for medians (*V*-values) on religiosity, fundamentalism, benevolent and hostile sexism toward women, right-wing authoritarianism, and social dominance orientation in our Main Study.

	Main effect Religious affiliation	Main effect Gender	Interaction effect Religion × Gender
Religiosity	*F*[2,315] = 154.66, *p* < 0.001^a,b,c,^ η^2^ = 0.45	*F*[1,315] = 0.44, *p* = 0.509, η^2^ = 0.00	*F*[2,315] = 0.65,*p* = 0.520, η^2^ = 0.00
Fundamentalism	*V*[2,∞] = 195.09, *p* < 0.001^a,b,c^	*V*[1,∞] = 0.91, *p* = 0.341	*V*[2] = 141.54,*p* < 0.001
Benevolent sexism	*F*[2,317] = 19.16, *p* < 0.001^b,c^, η^2^ = 0.11	*F*[1,317] = 5.50, *p* = 0.020, η^2^ = 0.03	*F*[2,317] = 0.43, *p* = 0.650, η^2^ = 0.00
Hostile sexism	*V*[2,∞] = 16.26, *p* < 0.001^b,c^	*V*[1,∞] = 13.72, *p* < 0.001	*V*[2] = 49.11,*p* < 0.001
Right-wing authoritarianism	*F*[2,325] = 15.10, *p* < 0.001^b,c^, η^2^ = 0.09	*F*[1,325] = 2.12, *p* = 0.146, η^2^ = 0.04	*F*[2,325] = 2.76,*p* = 0.065, η^2^ = 0.02
Social dominance orientation	*V*[2,∞] = 0.00,*p* = 0.999	*V*[1,∞] = 0.21, *p* = 0.649	*V*[2] = 3.39,*p* = 0.184

*Superscripted letters indicate which of the three post hoc group comparisons for the main effect for religious affiliation are significant at p < 0.05 after Bonferroni–Holm correction: a: Non-religious – Christian, b: Non-religious – Muslim, c: Christian – Muslim*.

An ANOVA conducted on religiosity revealed a main effect of religious affiliation but neither a main effect of gender nor an interaction effect. Non-religious participants showed the lowest levels of religiosity, Christians higher levels, and Muslims the highest (all pairwise comparisons *p* < 0.001).

Regarding fundamentalism, an ANOVA for medians revealed a main effect of religion but no effect of gender. The interaction was also significant. *Post hoc* analyses showed no difference between the genders among non-religious (*H* = 0.79, *p* = 0.375) and Christian participants (*H* = 2.24, *p* = 0.269), while male Muslims reported stronger fundamentalism than female Muslims (*H* = 20.31, *p* < 0.001). Non-religious participants reported lower fundamentalism than either Christians (*H* = 7.56, *p* = 0.006) or Muslims (*H* = 75.99, *p* < 0.001). The comparison between Christians and Muslims also revealed significant differences (*H* = 139.77, *p* < 0.001), with Muslims reporting stronger fundamentalism.

An ANOVA for benevolent sexism showed main effects for religious group and gender but no interaction effect. *Post hoc* analyses showed no difference between non-religious participants and Christians (*t*[73.15] = -1.43, *p* = 0.155), while both differed significantly from Muslims, who showed more benevolent sexism (compared to non-religious participants: *t*[67.93] = -6.27, *p* < 0.001; compared to Christians: *t*[246.49] = -6.67, *p* < 0.001). The gender effect was due to male participants reporting stronger benevolent sexism than female participants (*M*_female_ = 2.54, *SD*_female_ = 0.76; *M*_male_ = 2.99, *SD*_male_ = 0.72). Further, pairwise comparisons revealed that female Christians (whom we used as the reference group in our moderated mediation analysis) showed less benevolent sexism than female Muslims (*t*[166.79] = -4.42, *p* < 0.001) and male Muslims (*t*[172.65] = -7.86, *p* < 0.001), but did not differ from male Christian (*t*[17.93] = -1.95, *p* = 0.200), female non-religious (*t*[45.99] = 1.05, *p* = 0.602), and male non-religious (*t*[13.89] = 0.26, *p* = 0.797) participants.

An ANOVA for medians for hostile sexism revealed main effects for religion and gender as well as an interaction effect. Pairwise comparisons revealed that male Muslims were more hostile toward girls than all remaining groups (all *p* < 0.001), while none of the other five groups differed significantly from each other.

Analyzing RWA in an ANOVA, we found a main effect of religion but neither an effect of gender nor of an interaction between gender and religion. Those without religious affiliation (*M* = 2.14, *SD* = 0.93) and Christians (*M* = 2.10, *SD* = 0.70) did not differ from each other (*t*[62.23] = 0.28, *p* = 0.779) while Muslims (*M* = 2.74, *SD* = 0.75) more strongly endorsed RWA beliefs (compared to non-religious participants: *t*[55.42] = -3.93, *p* < 0.001; compared to Christians: *t*[229.31] = -7.33, *p* < 0.001).

Regarding SDO, there were no effects of religion, gender, or their interaction.

**FIGURE 1:**
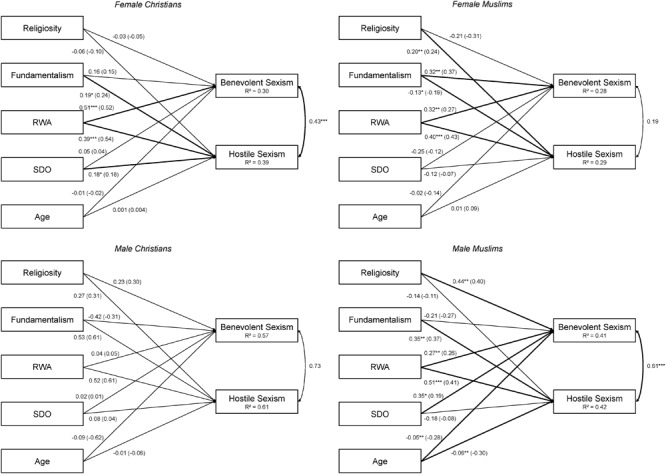
Path diagram of the multigroup multivariate regression analysis on hostile and benevolent sexism toward girls. Unstandardized and standardized regression weights (in parentheses) for female Christians, female Muslims, male Christians, and male Muslims in Main Study. ^∗^*p* < 0.05, ^∗∗^*p* < 0.01, ^∗∗∗^*p* < 0.001. All predictors were centered at their group-specific means.

We then estimated the multigroup multivariate regression (see Figure 1), only taking participants of Muslim or Christian faith into account. The bivariate correlation coefficients are reported in Supplementary Table S3 in the Appendix. Figure 1 illustrates the results for both benevolent and hostile sexism toward girls. Within the group of male Muslims, a higher degree of self-reported religiosity was significantly associated with higher degrees of benevolent sexism. Additionally, for female Muslims higher levels of fundamentalism were associated with higher levels of benevolent sexism. RWA predicted benevolent sexism significantly in all groups, except for male Christians. Only among male Muslims was SDO an additional positive predictor and age an additional negative predictor of benevolent sexist attitudes toward girls.

For hostile sexism, religiosity was predictive only among female Muslims. At the same time, female Muslims were less hostile toward girls the stronger they expressed fundamentalist beliefs. In the remaining three groups, hostile sexism increased with fundamentalism, albeit not significantly so for male Christians. RWA was a strong predictor of hostility in all four groups but, again, not significantly so for male Christians. SDO emerged as an additional predictor for female Christians. As was the case for benevolent sexism, the older Muslim participants were, the less they endorsed hostile sexism, whereas age did not have an effect in any of the remaining groups.

We then conducted the moderated mediation analysis specifying female Christians as the reference group. Results are depicted in Table 7. For benevolent sexism toward girls, in the analysis for male Christians TE was statistically significant but the indirect and direct effects were not. While falling short of the significance threshold may have been due to the extremely small sample size, results seem to suggest that the stronger benevolent sexism of male Christians as compared to female Christians, indicated by the significant TE, cannot be explained by any of the mediating variables. When comparing male Christians to female Christians in their levels of hostile sexism, a similar picture emerged. In this case even TE was not statistically significant.

**Table 7:** Results of the moderated mediation analysis predicting benevolent and hostile sexism toward girls: medians and 97% Confidence Intervals (in parentheses) generated by the Monte-Carlo resampling approach in Main Study.

	Total natural indirect effect			Pure natural direct effect	Total effect
Group^a^	Religiosity	Fundamentalism	All mediators^b^		
**Benevolent sexism**					
Female Muslims	–0.19 (–0.44; 0.03)	0.48^∗∗^ (0.18; 0.81)	0.40^∗∗^ (0.13; 0.66)	0.08 (–0.21; 0.38)	0.48^∗∗∗^ (0.27; 0.70)
Male Christians	0.00 (–0.23; 0.36)	0.06 (–0.08; 0.34)	–0.00 (–0.35; 0.59)	0.42 (–0.06; 0.91)	0.42^∗^ (0.03; 1.08)
Male Muslims	0.34^∗∗^ (0.10; 0.62)	–0.45 (–0.94; 0.04)	0.25 (–0.14; 0.65)	0.53^∗^ (0.09; 0.98)	0.78^∗∗∗^ (0.59; 0.98)
**Hostile sexism**					
Female Muslims	0.18^∗∗^ (0.04; 0.34)	–0.20^∗^ (–0.37; -0.04)	0.14 (–0.02; 0.32)	–0.09 (–0.26; 0.09)	0.05 (–0.10; 0.21)
Male Christians	0.01 (–0.23; 0.55)	–0.08 (–0.35; 0.09)	–0.02 (–0.47; 0.94)	0.52 (–0.07; 1.04)	0.51 (–0.03; 1.36)
Male Muslims	–0.11 (–0.41; 0.15)	0.74^∗∗^ (0.27; 1.24)	1.22^∗∗∗^ (0.78; 1.69)	0.60^∗^ (0.11; 1.09)	1.82^∗∗∗^ (1.60; 2.04)

*^*a*^Female Christians are used as the reference group in this analysis. ^*b*^This is the combination of the indirect effects of religiosity, fundamentalism, SDO, and RWA.^∗^ p < 0.05, ^∗∗^ p < 0.01, ^∗∗∗^ p < 0.001*.

For female Muslims, in contrast, the stronger benevolent sexism we found, as compared to female Christians, was mediated by fundamentalism: female Muslims were more in favor of fundamentalist religious beliefs, and this was accompanied by stronger levels of benevolent sexism. In this analysis, both TNIE and TE were statistically significant. The higher level of religiosity found for female Muslims, as compared to female Christians, was associated with higher levels in hostile sexism. The opposite was the case for fundamentalism, where the higher levels were accompanied by less hostile sexism toward girls. Overall, this resulted in a non-significant TE, as female Muslims did not differ from female Christians in their hostile sexism toward girls (Table 5).

For male Muslims, their more pronounced benevolent sexism, as compared to female Christians, was partly mediated by their stronger religiosity, accompanied by a significant PNDE. The significant PNDE suggests that male Muslims approved more strongly of benevolent sexism toward girls than female Christians did to an extent beyond what can be explained by our mediator variables. A slightly different pattern emerged for hostile sexism, where male Muslims’ stronger hostility, as compared to female Christians, was partly mediated by their more pronounced fundamentalism. As for benevolent sexism, a significant PNDE emerged even after inclusion of the two religion-related variables, RWA, SDO, and age, suggesting that male Muslims differed from female Christians in their hostility toward girls due to factors not covered by our analysis.

To summarize, the results of our mediated moderation analysis show that the stronger benevolent and hostile sexism we observed in male Muslims (as compared to female Christians) can be partly, but not completely, explained by religiosity and fundamentalism. However, religiosity and fundamentalism mediated the differences in ambivalent sexism we found between female Muslims and female Christians. In this case, once our mediating variables were taken into account, female Muslims no longer showed PNDEs in their ambivalent sexism when compared to female Christians. The comparison between male Christians and female Christians, in contrast, did not indicate mediation by religion or fundamentalism. Thus, in this case the stronger endorsement of benevolent sexism we observed in TE for male participants cannot not be explained by religiosity or fundamentalism.

## General Discussion

In this research, we sought to investigate whether religiosity and fundamentalism as such, rather than specific religious affiliation, would be predictors of ambivalent sexism toward girls and women.

We further aimed to disentangle the interrelations between religion- and sexism-related variables, while taking into account two variables that previous research suggested as potential confounders but had not been included in prior investigations: right-wing authoritarianism and social dominance orientation.

### Religiosity and Fundamentalism

In all three studies, Muslim participants described themselves as more religious than Christian participants, who, in turn, described themselves as more religious than participants without religious affiliation. The same pattern was observed for fundamentalism. Muslims held more fundamentalist religious beliefs than Christians, who held more such beliefs than non-religious participants. These differences between religious groups were independent of participants’ gender and replicated the findings of previous research (Heitmeyer et al., 1997; Brettfeld and Wetzels, 2007; Frindte et al., 2011; Huber and Huber, 2012; de Hoon and van Tubergen, 2014; Koopmans, 2015; Gille, 2016).

### Interrelations Between Religious Affiliation, Religion, Fundamentalism, and Ambivalent Sexism

To the best of our knowledge, no previous research has compared the relation between religious affiliation and sexist attitudes on either a national or international level. In both Pilot Studies as well as our Main Study, Muslim participants approved of benevolent sexism toward girls and women more than Christian and non-religious participants, with the latter two groups showing no difference from one another. Regarding hostile sexism, our findings were somewhat inconsistent. While in our first Pilot Study investigating attitudes toward girls the three religious and non-religious groups did not differ from each other in their levels of hostile sexism, in our second Pilot Study Muslims approved of hostile attitudes toward women more strongly than the other two groups, and in our Main Study male Muslims endorsed hostile attitudes toward girls more strongly than female Muslims, male Christians and female Christians, who did not differ from one another. We do not know whether the findings of our Main Study would have been replicated if we had reached a larger sample of male Muslims and/or if we had included the potential confounders RWA and SDO in our first Pilot Study.

The stronger ambivalent sexism indicated by our Muslim participants corresponds to the higher levels of benevolent and hostile sexism that previous studies have found in Muslims living in Muslim-majority countries (Taşdemir and Sakallı-Uğurlu, 2010; Glick et al., 2016) compared to Christians living in Christian-majority countries (Glick et al., 2002; Maltby et al., 2010; Mikołajczak and Pietrzak, 2014). Our findings show that, on average, Muslims living in Germany endorse higher levels of ambivalent sexism than the Christian majority group, despite many of them being third- or fourth-generation residents of Germany (for similar findings regarding other dependent measures see for instance Diehl and Koenig, 2009; Stanat et al., 2010; Jacob and Kalter, 2013; Walter, 2014). These findings could possibly indicate that members of the Muslim minority in Germany feel discriminated against, thereby fueling reactive ethnicity and the adoption of acculturation strategies of separation rather than assimilation (cf. Phinney et al., 2006; Verkuyten and Yildiz, 2007).

Results from our moderated mediation analysis suggest that differences in ambivalent sexism between the two religious groups were partly due to religiosity, fundamentalism, RWA, and SDO. More specifically, the religiosity-sexism link reported by previous research was replicated in all of our studies in medium-sized bivariate correlations, with religious participants showing stronger benevolent and hostile sexism toward girls and women. However, when differentiating participants according to religious affiliation and gender in the multigroup multivariate regression analysis in our Main Study, a more complex picture emerged.

As previous research has found fundamentalism (e.g., Banyasz et al., 2016; Harnish et al., 2017) and ambivalent sexism (Sibley et al., 2007) to be correlated with RWA and/or SDO, we included both ideologies in our analyses. While not significant in our small sample of male Christians, we found that RWA was strongly associated with hostile sexism in all four groups, i.e., irrespective of participants’ gender and religious affiliation. The strong correlation between RWA and hostile sexism is in line with previous research that has shown RWA to predict prejudice and hostility in a wide range of intergroup relations (e.g., McFarland, 1989; Hunsberger et al., 1999; Nagoshi et al., 2008; Whitley, 2009; Hall et al., 2010; Hill et al., 2010). In addition, participants (with the exception of female Muslims) showed more hostile sexism toward girls the more fundamentalist their religious beliefs were (this prediction was, again, not statistically significant in our small sample of male Christians). Interestingly, once fundamentalism was accounted for, religiosity did not contribute to the prediction of hostile sexism (except for the group of female Muslims who reported more hostility toward girls the more religious they described themselves to be^5^). While there were some differences between subgroups, these findings seem to suggest that fundamentalism was more important for the prediction of hostile sexism than religiosity.

A quite different picture appeared for benevolent sexism toward girls, where RWA proved to be a significant predictor in all groups but male Christians. In our Christian subsamples, no variable aside from RWA contributed to the prediction of benevolent sexism. In contrast, the religion-related variables explained variance in benevolent sexism among our Muslim participants: benevolent sexism increased with religiosity in male Muslims and with fundamentalism in female Muslims.

These findings suggest that approval of traditional values and obedience toward authority figures, as measured by participants’ endorsement of RWA (Altemeyer, 1996), predict hostile and benevolent sexist attitudes toward girls irrespective of religious affiliation. There may be a relation between allegiance to traditional values and the approval of the status-quo gender hierarchy as well as between avowal of obedience toward authority figures and approval of female submission to male family members. Interestingly, in our Christian subsamples variations in benevolent sexism were only dependent on RWA, whereas in our Muslim subsamples religiosity and fundamentalism mattered as well. This finding suggests that there are specific contents of the religious teachings of Islam which encourage benevolent sexism and are not fully captured by the approval of conventionalism and authoritarian submission (as measured by RWA).

Results of the moderated mediation analysis conducted in our Main Study suggest that the differing degrees of ambivalent sexism between female Muslims and female Christians were explained by our mediating variables, in particular by female Muslims’ stronger fundamentalism. The stronger (as compared to female Christians) benevolent and hostile sexist attitudes that male Muslims indicated having toward girls were partly mediated by the religion-related variables. More specifically, highly religious boys and men approved more strongly of benevolent sexist propositions. Benevolent sexism is supposed to reward girls and women who adhere to their traditional role. It is possible that highly religious boys and men hold particularly traditional views on the female role and are thus also more inclined to see girls and women as “wonderful” and in need of male protection, as stipulated in the conceptualization of benevolent sexism (cf. Glick and Fiske, 1996, 2001). The difference in hostile sexism between male Muslims and female Christians was attributable to male Muslims’ stronger fundamentalism. By including RWA and SDO when predicting hostile sexism, we accounted for the potential influence of cognitively rigid thinking (Hill et al., 2010), traditionalism and authoritarianism (Brandt and Reyna, 2014), strivings for dominance, and negative attitudes toward individuals violating in-group norms (Sibley et al., 2007). Our finding that fundamentalism in male Muslims additionally contributed to the prediction of hostile sexism toward girls suggests that fundamentalism captures features other than those attributed to RWA and SDO, features, moreover, that are unique to religion-related ideology. Our mediation analyses for male Muslims indicated a direct effect for both benevolent and hostile sexism, even after religiosity, fundamentalism, RWA, and SDO had been accounted for. Hence, there are factors other than those covered by our model that are responsible for their stronger ambivalent sexism.

This complex pattern of findings calls for future research examining the association of religion and ambivalent sexism in larger samples from different religious affiliations. In particular, larger samples of male Christians need to be investigated as in both our studies that included data from online surveys (Pilot Study 1, Main Study) this group was clearly underrepresented as compared to Christian girls and women. Possibly, this asymmetry was abetted by the fact that girls and women are overrepresented among Christians in Germany (55% of church members are female), in particular among active church members who, for instance, volunteer in church work (74% girls and women), perform official duties in their local church (1.7% of the female and 0.4% of the male church members), or are employed by the church (in positions other than priests 80% of the employees are women; all statistics from Studienzentrum der Ekd für Genderfragen, 2015). Christian girls and women being more committed to their religion than Christian boys and men could imply that male Christians show a lower willingness to participate in surveys about their values and religion on a voluntary basis than female Christians do. Future studies should also include controls for immigrants’ ethnic or cultural background, the number of generations their families have been living in the host country, and their highest completed level of education.

We started by citing anecdotal evidence linked to claims made by the general public that Muslim men were particularly sexist toward girls and women. We then tested whether the higher religious self-identification and stronger endorsement of fundamentalism among Muslim participants in comparison to non-religious and Christian participants offered a more precise explanation of differences in ambivalent sexism than simply belonging to a specific religion, namely Islam. While our studies have provided initial evidence that stronger religiosity and fundamentalism explain some of the variance in ambivalent sexism, these varying levels of religious involvement cannot entirely explain the particularly strong hostile and benevolent sexism we found in male Muslims. Hence, there are factors responsible for their stronger ambivalent sexism other than those investigated in our studies. Glick et al. (2016) have suggested that these factors may include specific contents of Islamic religious teachings. “The Qur’an,” they write, “includes verses that seem to offer both subjectively hostile and benevolent justifications for gender hierarchy. On the hostile side, the Qur’an calls for women to submit to men as their inferiors… On the subjectively benevolent side, the Qur’an instructs men to protect and provide for women” (p. 546).

An additional factor that might explain the stronger ambivalent sexism we found in our Muslim male participants are honor beliefs. Muslim-majority nations with the largest numbers of Muslim immigrants living in Germany (Turkey and member states of the Arab league) have been described as “cultures of honor” (Nisbett and Cohen, 1996). This term refers to collectivistic cultures that emphasize the value of social reputation, which is frequently associated with prescribed gender-specific behaviors supportive of male power and female subordination. While men gain honor through strength and aggression, women are recognized for sexual purity and obedience toward male family members (Vandello and Cohen, 2003). Men are expected to defend the honor of their family even if it involves using force or punishing women for disobedient behavior, but they are also expected to provide for women and behave chivalrously toward them. Accordingly, Glick et al. (2016) found honor beliefs in men to correlate particularly strongly with hostile sexism and moderately strongly with benevolent sexism. It is therefore likewise possible that the stronger hostile and benevolent sexism we found in male Muslims after we had controlled for religiosity, fundamentalism, RWA, SDO, and age, was due to their stronger honor beliefs.

What can be learned from our findings with respect to the prevention of sexism toward girls and women? Right-wing authoritarianism and religious fundamentalism proved to be strongly correlated with ambivalent sexism irrespective of our participants’ religious orientation and contributed to an explanation of the particularly strong hostile sexism toward girls that we have found among our sample of Muslim boys and men in our Main Study. Democratic institutions, such as schools or universities, as well as religious institutions, should strengthen their efforts to diminish the influence of fundamentalist beliefs by teaching the right to freedom of expression and the right to dissent. By promoting tolerance and reasonableness, we can counter the misuse of religion to discriminate against girls and women and promote gender equality in multireligious societies.

## Ethics Statement

According to our institution’s guidelines and national regulations, no ethics approval was required, since our research used anonymous or no-risk tests, surveys, interviews, or observations. In this study no private information (such as name, address, IP address or email address) was collected. Participants’ consent was obtained by virtue of survey completion.

## Datasets Are Available on Request

The raw data supporting the conclusions of this manuscript will be made available by the authors, without undue reservation, to any qualified researcher.

## Author Contributions

BH provided the initial idea for the study. BH, LZ, and JG contributed to the conception and design of the study. JG and LZ organized, conducted the data collection, and organized the database. MS performed the statistical analysis. BH wrote the first draft of the manuscript. BH and MS wrote the manuscript’s method section. All authors contributed to manuscript revision, read and approved the submitted version.

## Conflict of Interest Statement

The authors declare that the research was conducted in the absence of any commercial or financial relationships that could be construed as a potential conflict of interest.

## Footnotes

1 Shia-Forum

2 religionsforum.de

3 youthweb.net

4 We altered the implementation of the RMediation-package to provide medians rather than means as the point estimates for effects.

5 As indicated by the bivariate correlation coefficients, hostile sexism was positively related to fundamentalism, religiosity, and RWA in all three studies as well as across all examined subsamples. Once we controlled for all these interrelated variables, the effect of fundamentalism on hostile sexism became negative and the effect of religiosity on hostile sexism became positive in one of our subsamples: female Muslims. We do not wish to interpret this deviation from the pattern of our findings until it has been replicated in future research.
