# Supplementary material for: Religiosity, Religious Fundamentalism, and Ambivalent Sexism Toward Girls and Women Among Adolescents and Young Adults Living in Germany
Source: Front Psychol. 2018 Dec 3;9:2399. doi: 10.3389/fpsyg.2018.02399 (PMC6286999; doi:10.3389/fpsyg.2018.02399)
Supplement: Supplementary file 1 [file Data_Sheet_1.PDF]

Supplementary Material

Religiosity, religious fundamentalism, and ambivalent sexism toward girls and women among adolescents and young adults living in Germany

Bettina Hannover*, John Gubernath, Martin Schultze, Lysann Zander

Correspondence: Dr. Bettina Hannover: bettina.hannover@fu-berlin.de

1 Supplementary Tables

Supplementary Table 1

Means and Standard Deviations (in Parentheses) for Religiosity, Fundamentalism, Benevolent and Hostile Sexism towards Girls Separated by Religious Groups and Participant Gender in Pilot Study 1

	Nonreligious			Christian			Muslim		
	Female (n = 16)	Male (n = 12)	Overall (n = 28)	Female (n = 32)	Male (n = 24)	Overall (n = 56)	Female (n = 8)	Male (n = 7)	Overall (n = 15)
Religiosity	2.00 (1.01)	1.88 (0.68)	1.95 (0.87)	3.37 (1.05)	3.53 (0.97)	3.44 (1.01)	4.52 (0.37)	4.55 (0.13)	4.54 (0.28)
Fundamentalism	1.86 (0.91)	1.50 (0.72)	1.71 (0.84)	2.25 (1.09)	2.61 (1.15)	2.40 (1.12)	4.02 (0.56)	4.14 (0.54)	4.08 (0.54)
Benevolent Sexism	2.59 (0.86)	3.25 (0.76)	2.87 (0.87)	2.57 (0.69)	3.32 (0.65)	2.89 (0.76)	3.19 (0.63)	4.01 (0.37)	3.57 (0.66)
Hostile Sexism	1.97 (0.69)	2.23 (0.94)	2.08 (0.80)	2.16 (0.72)	2.45 (0.77)	2.29 (0.75)	2.33 (0.61)	2.68 (0.65)	2.49 (0.63)

Note. All items were measured on 1–5 response scales, with higher values indicating stronger endorsement.

Supplementary Table 2

Means and Standard Deviations (in Parentheses) for Religiosity, Fundamentalism, Benevolent and Hostile Sexism towards Women, Right-Wing Authoritarianism, and Social Dominance Orientation Separated by Religious Groups and Participant Gender in Pilot Study 2

	Nonreligious			Christian			Muslim		
	Female (n = 28)	Male (n = 24)	Overall (n = 52)	Female (n = 19)	Male (n = 15)	Overall (n = 34)	Female (n = 19)	Male (n = 29)	Overall (n = 48)
Religiosity	1.72 (0.78)	1.40 (0.58)	1.57 (0.71)	2.47 (1.09)	2.57 (1.42)	2.51 (1.23)	3.55 (0.98)	3.84 (0.88)	3.72 (0.92)
Fundamentalism	1.19 (0.34)	1.25 (0.44)	1.21 (0.39)	1.60 (1.02)	1.72 (0.89)	1.65 (0.95)	3.27 (1.36)	3.65 (1.19)	3.50 (1.25)
Benevolent Sexism	2.25 (1.05)	2.88 (1.12)	2.54 (1.12)	2.85 (1.29)	2.84 (1.15)	2.85 (1.21)	4.36 (1.24)	4.37 (1.19)	4.36 (1.20)
Hostile Sexism	1.76 (0.91)	2.08 (0.90)	1.91 (0.91)	1.98 (1.02)	2.06 (0.91)	2.01 (0.96)	3.33 (0.96)	3.42 (1.20)	3.38 (1.10)
Right-Wing Authoritarianism	1.88 (0.74)	2.01 (0.67)	1.94 (0.70)	2.04 (0.72)	2.15 (0.79)	2.09 (0.74)	3.09 (0.97)	3.08 (0.86)	3.09 (0.90)
Social Dominance Orientation	1.68 (0.49)	1.90 (0.67)	1.78 (0.59)	1.66 (0.58)	2.03 (0.70)	1.83 (0.66)	2.43 (0.63)	2.53 (0.82)	2.49 (0.75)

Note. Religiosity, fundamentalism, RWA, and SDO measures used 1–5 response scales; benevolent Sexism and Hostile Sexism were measured on 1–6 response scales. Higher values indicate stronger endorsement.

Supplementary Table 3

Correlations among Religiosity, Religious Fundamentalism, Benevolent and Hostile Sexism towards Girls, Right-Wing Authoritarianism, and Social Dominance Orientation in Main Study

	Religiosity	Fundamentalism	Benevolent Sexism	Hostile Sexism	Right-Wing Authoritarianism
Religiosity	-				
Fundamentalism	.68***<br>(.67*** / .23 / .51*** / .58***)	-			
Benevolent Sexism	.38***<br>(.18 / .35 / -.02 / .37**)	.49***<br>(.27 / .06 / .38 / .18)	-		
Hostile Sexism	.28***<br>(.20 / .23 / .13 / .11)	.55***<br>(.31* / .70 / .04 / .49***)	.53***<br>(.62*** / .59 / .23 / .52***)	-	
Right-Wing Authoritarianism	.27***<br>(.25 / .02 / .02 / .01)	.52***<br>(.29 / .66 / .38** / .32*)	.50***<br>(.55*** / .33 / .41 / .20***)	.60***<br>(.58*** / .76* / .35** / .51***)	-
Social Dominance orientation	.09<br>(.13 / -.45 / .27 / .09)	.06<br>(-.02 / -.23 / .21 / .12)	-.01<br>(.06 / -.53 / -.05 / .11)	-.04<br>(.18 / -.47 / -.06 / -.08)	-.02<br>(.05 / -.54 / .00 / .01)

Notes. Correlations and *p*-values for each subsample are shown in parentheses in the order female Christians/ male Christians / female Muslims/ male Muslims. Due to occasional missing data, *N*s range as follows: 309 – 326 (88 – 91 / 12 – 15 / 85 – 90 / 81 – 89). *p*-values were Bonferroni-Holm corrected within each group but not across groups. * $p < .05$, ** $p < .01$, *** $p < .001$.
